# Life history strategy of *Tubastraea* spp. corals in an upwelling area on the Southwest Atlantic: growth, fecundity, settlement, and recruitment

**DOI:** 10.7717/peerj.17829

**Published:** 2024-07-31

**Authors:** Nathália Bastos, Layla Poubel Tunala, Ricardo Coutinho

**Affiliations:** 1 Departament of Marine Biotechnology, Instituto de Estudos do Mar Almirante Paulo Moreira–IEAPM, Arraial do Cabo, Rio de Janeiro, Brasil; 2 Department of Geosciences, Postgraduate Program in Ocean and Earth Dynamics–DOT, Universidade Federal Fluminense–UFF, Niterói, Rio de Janeiro, Brasil

**Keywords:** Bioinvasion, Larval settlement, Sun-coral reproduction, Arraial do Cabo

## Abstract

Over the past few decades, corals of the genus *Tubastraea* have spread globally, revealing themselves to be organisms of great invasive capacity. Their constant expansion on the Brazilian coast highlights the need for studies to monitor the invasion process. The growth, fecundity, settlement, and data on the coverage area of three co-occurring *Tubastraea* species in the 2015–2016 period were related to temperature variation and light irradiance on the rocky shores of Arraial do Cabo, Rio de Janeiro. Hence, this study sought to understand and compare the current invasion scenario and characteristics of the life history strategy of sun coral species based on environmental variables, considering the uniqueness of this upwelling area in the southwestern Atlantic. For that, we evaluate the fecundity, settlement, and growth rates of corals by carrying out comparative studies between species over time and correlating them with the variables temperature and irradiance, according to seasonality. Field growth of colonies was measured every two months during a sample year. Monthly collections were performed to count reproductive oocytes to assess fecundity. Also, quadrats were scrapped from an area near a large patch of sun coral to count newly attached coral larvae and used years later to assess diversity and percentage coverage. Results showed that corals presented greater growth during periods of high thermal amplitude and in months with below-average temperatures. Only *Tubastraea* sp. had greater growth and polyp increase in areas with higher light incidence, showing a greater increase in total area compared to all the other species analyzed. Despite the observed affinity with high temperatures, settlement rates were also higher during the same periods. Months with low thermal amplitude and higher temperature averages presented high fecundity. While higher water temperature averages showed an affinity with greater coral reproductive activity, growth has been shown to be inversely proportional to reproduction. Our study recorded the most significant coral growth for the region, an increase in niche, high annual reproductive activity, and large area coverage, showing the ongoing adaptation of the invasion process in the region. However, lower temperatures in the region affect these corals’ reproductive activity and growth, slowing down the process of introduction into the region. To better understand the advantages of these invasion strategies in the environment, we must understand the relationships between them and the local community that may be acting to slow down this colonization process.

## Introduction

Capable of colonizing and establishing themselves in environments far from their natural range, some scleractinians of the genus *Tubastraea* Lesson, 1829 are found worldwide living in natural and artificial substrates of shallow tropical waters (Cairns, 1994). These corals have expanded their distribution in the western Atlantic in the last three decades, becoming increasingly common in the underwater landscapes of the Gulf of Mexico, Caribbean and Brazil (Creed et al., 2016; Sammarco et al., 2015; Fenner, 2001). Originally described as native to the Indo-Pacific, the coral *Tubastraea coccinea* Lesson, 1830 is well-established on the tropical and sub-tropical western Atlantic benthic communities, at times dominating certain areas (Sammarco et al., 2017). Together with *Tubastraea tagusensis* Wells, 1982 (De Paula & Creed, 2004), originally from Galápagos, they occur discontinuously over 3,000 km along the Brazilian coast (Creed et al., 2016), and Gulf of Mexico (Figueroa et al., 2019). In Brazil, both species were introduced into the south-western Atlantic on oil platforms (De Paula & Creed, 2005) and were first observed in natural substrates in the 1990s, in the regions of Ilha Grande Bay and Arraial do Cabo/RJ (De Paula & Creed, 2004; Ferreira, 2003). Since then, these corals have been expanding their distribution along the entire coastline (Silva et al., 2011; Mantelatto et al., 2011; Sampaio et al., 2012; Costa et al., 2014; Creed et al., 2017; Soares et al., 2020), occupying areas with different species compositions (Vançato, Creed & Fleury, 2023).

*Tubastraea* invasions own their success mostly to the genus’s highly competitive potential which allows for rapid expansion and colonization of new areas (Cairns, 2000; Sammarco, Atchison & Boland, 2004). With great invasive fitness, efficient reproductive strategies, ability to settle in different substrates, high growth rate, rapid species establishment, fast dominance of new areas, and high dispersal ability (Vermeij, 2005; Creed & De Paula, 2007; Luz et al., 2020), *Tubastraea* corals have high recruitment and settlement rates, resulting in a potential to occupy empty substrates (De Paula & Creed, 2005; Mizrahi, 2008; Mizrahi, Navarrete & Flores, 2014) and rapid growth rates (Vermeij, 2005; Lages et al., 2011; De Paula, 2007). The species *Tubastraea coccinea* has hermaphroditism, external fertilization (Ayre & Resing, 1986) or self-fertilization, broods and releases lecithotrophic larvae and has continuous reproduction (Fenner & Banks, 2004; Glynn et al., 2008; De Paula, Pires & Creed, 2014). It was recorded producing asexual planulae in Australia (Ayre & Resing, 1986), and was described performing self-fertilization in Brazil by De Paula, Pires & Creed (2014). *Tubastraea aurea* (formerly synonymized as *T. coccinea*) was reported as a gamete-releasing coral on the Great Barrier Reef, Australia (Harrison, 1985). It showed larval longevity of more than 91 days and an extended period of competence of 69 days (Luz et al., 2020). According to Vermeij (2006), the onset of reproductive age occurs at 1.5 years, but Glynn et al. (2008) state that *Tubastraea* corals mature early, presenting reproductive age with only two polyps.

The species *T. coccinea* and *T. tagusensis* represent the first introduction of scleractinian corals in the South Atlantic (De Paula & Creed, 2004). Ecological studies and new records commonly refer to species previously described for Brazil by De Paula & Creed (2004) without a new taxonomic assessment. Currently, different morphotypes of sun coral can be seen all along the Brazilian coast, and in Arraial do Cabo a recent study evaluating morphology integrated with molecular data described the occurrence of three morphotypes—*T. aurea*, *T. coccinea*, and *Tubastraea* sp. (Bastos et al., 2022). This study considered all three morphotypes as species because even though *T. aurea* did not show molecular support, by the ITS marker, to validate it as a species, it did show enough statistical support to differentiate it morphologically and therefore, demonstrates particular biological attributes. Importantly, the species *T. tagusensis* identified for Brazil through samples from Ilha Grande Bay, approximately 230 km to the south of the study area, was not identified for Arraial do Cabo due to morphological divergences. Contrary to projections described in the literature for invasive corals, in which mainly *T. coccinea* has successful colonization and an increase in abundance is expected over time, research suggests a more restricted development of sun corals in Arraial do Cabo (Araújo, 2023), especially when compared to the invasion pattern in other locations, such as Ilha Grande Bay (Creed et al., 2021; De Paula & Creed, 2004), the coast of São Paulo (Mizrahi et al., 2017), the Gulf of Mexico (Sammarco et al., 2017), and the coast of Florida (Fenner & Banks, 2004). Despite occurring at almost every dive site within the bay, they do not dominate the landscape and, according to Batista et al. (2017), the causes of this restricted expansion are the lower average temperatures and the high diversity of benthic communities (Araújo, 2023) in Arraial do Cabo.

Species success in the invasion process depends on many factors, and fecundity and growth are the main characteristics that determine the capacity of invading species (Harrison & Wallace, 1990). Growth rate-along with the settlement rate-can indicate potential coral expansion and provide a better understanding of population dynamics and the behavior of invaders. The number or frequency of individuals, eggs or larvae and the larvae dispersion are predominantly related to success in the colonization process (Gaines & Bertness, 1993) and fecundity is a key demographic process for population dynamics studies (Álvarez-Noriega et al., 2020). Information on the biology of sun corals is important for defining control strategies. Studies of reproduction and larval biology are important to better understand the life history and ecology of scleractinian populations (Fadlallah, 1983; Harrison & Wallace, 1990). In this regard, reproduction studies help to develop control measures for invasive populations (Wotton et al., 2004). Knowledge of the ecological and biological aspects of these corals is fundamental for environmental and biota management.

To understand the life history strategies of three *Tubastraea* species that co-occur in Arraial do Cabo, based on life history characteristics, this study addressed the following questions: 1) What is the growth rate of corals?; 2) What is the fecundity rate?; 3) What are the settlement rates?; 4) What is the coverage area?; and considering the external environmental factors, 5) How do temperature and luminosity affect these parameters? This study adds new information on the processes that control their dispersion in the region of Arraial do Cabo, considering the particularities of this environment, a marginal site limiting the distribution of many marine species. We offer evidence of extensive seasonal reproductive activity, high fecundity, presence of settled larvae almost all year round and colonies formed from successful recruitment. These attributes contribute to the prominence of *Tubastraea* on the rocky shores in a region of high ecological value. The biological parameters evaluated allow us to understand the species’ establishment processes, their reproductive, growth, and recruitment aspects, as well as to evaluate this dynamism in distinct irradiance environments. Understanding population dynamics is essential for predicting increased distribution and tolerance to environmental variations of these corals.

## Materials and Methods

Portions of this text were previously published as part of a thesis (https://app.uff.br/riuff/bitstream/handle/1/24815/66%20-%20TESE%20-%20Nathalia%20Bastos%202020..pdf?isAllowed=y&sequence=1).

### Study area

**Figure 1:**
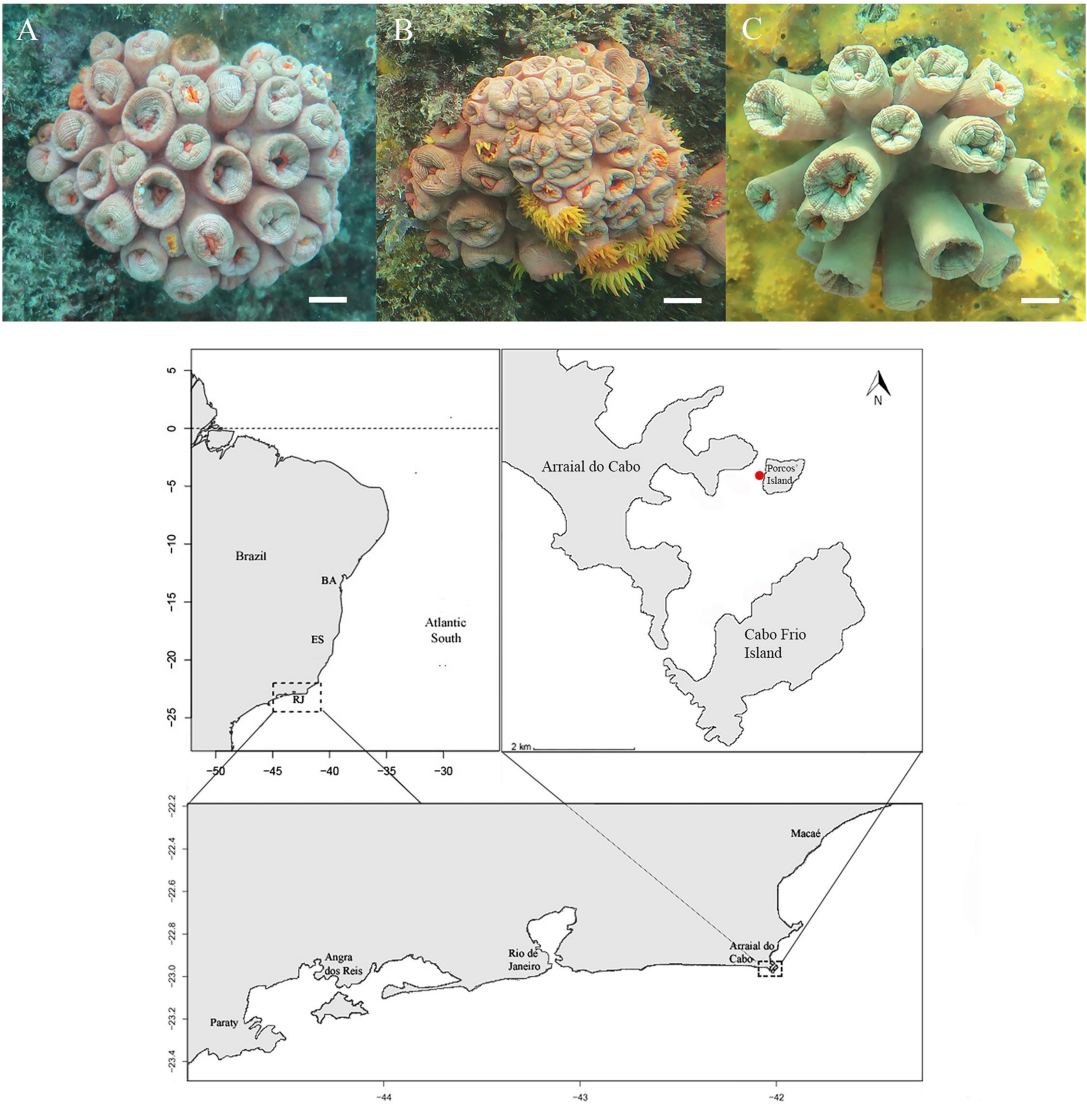
Morphotypes identification of Tubastraea genus and location. (A and B) *T. coccinea* species (A–*T. aurea* synonymy of *T. coccinea*, B–*T. coccinea*), and (C) *Tubastraea sp.* at the top of the image and the sample location indicating Arraial do Cabo (RJ) in southwest of Brazil.

Arraial do Cabo, located at Rio de Janeiro State (22°57′ S–42°1′ W), is a transition zone between the tropical and warm temperate Southwest Atlantic Ocean (Spalding et al., 2007) and is influenced by an upwelling system. Despite being a tropical region on the Brazilian coast, Arraial do Cabo has recorded many occurrences of low temperatures (Batista et al., 2017). According to Valentin (1984b), in coastal geography, the prevailing easterly and north-easterly winds result in an elevation of cold water filled with nutrients that enrich primary production. Arraial do Cabo Bay is a sheltered area, protected from the direct action of upwelling systems and oceanic waves (Fig. 1). During the non-upwelling period, coastal surface waters are characterized by temperatures above 21 °C, in conditions of upwelling by low temperature values below 18 °C, which can reach 13 °C (Guimaraens & Coutinho, 1996). Due to these peculiar characteristics, Arraial do Cabo has a unique environment, with rocky shores that support a highly diverse underwater benthic community, and is also considered the southern tropical limit for many species, including corals (Laborel, 1969; Castro & Pires, 2001; Ferreira, Gonçalves & Coutinho, 2001). Due to the expansion in offshore oil exploration, Porto do Forno, located in Arraial do Cabo Bay, began to house platforms and supplier activities, thus promoting the arrival and establishment of nonnative species (Ferreira, Gonçalves & Coutinho, 2006), which currently operate in other port activities. This port does not currently carry out biological invasion control (IBAMA, 2018).

Porcos Island—the sampling site for this study-borders Arraial do Cabo Bay to the North (Candella, 2009). The sampled side of the island is subject to little wave action and is protected from the prevailing NE winds (Carriére et al., 2009). Few records of cold water below 15 °C and high temperatures ranging from 25 to 27 °C near the study area were found in Batista et al. (2017), creating environmental conditions for an adequate calcification rate of azooxanthellate and zooxanthellate corals (Marshall & Clode, 2004). This region houses a tropical coral oasis (Laborel, 1969) with a substrate displaying rocks of various diameters and heights that extends from the intertidal zone to a sandy plain 5 to 13 m deep. The subtidal benthic community consists of anthozoans, ascidians, polychaetes, algae, sponges, bryozoans, and barnacles (Ferreira, 2003; Araújo, 2023). This site holds the highest density of sun coral colonies among the sites studied by Batista et al. (2017), with 98 colonies per 15 m^2^, and the highest density of recruits, with 44 per 15 m^2^. According to the authors, they probably comprise the first populations of *Tubastraea* corals in Arraial do Cabo Bay.

### Experimental design

#### Growth

Growth data of three species were collected during a one-year sampling period, from November 2015 to October 2016, by scuba diving. The colonies were chosen at random, constituting the average occurrence of adult sizes, and marked with an identification tag in the field. Total sample was divided into two groups of 20 colonies each (*N* = 40), for each species (N_total_ = 120), according to their positions in areas of high or low light irradiance (HL and LL, respectively). HL and LL areas were defined by visual observation according to substrate slope, but previous information for the same location and depths on irradiance rates were provided by Tunala et al. (2019). The sites defined as LL were small caves and rocks with negative slopes. HL sites were less slopped areas or rocks exposed directly to light. Forty individuals of each species had their largest and smallest diameters measured using a caliper ruler during scuba diving at depths between 3 and 5 m, in places with a high density of *Tubastraea* species. Measurements were taken once every two months (T0 = first measurement and T5 = last measurement). Colony area (A), *i.e*., the coverage area, was calculated using A = (d_major_/2) × (d_minor_/2) × 3.14, in which d_major_ is the largest diameter and d_minor_ is the smallest diameter of the colony in centimeters (*e.g*., Connell, 1973; Hughes & Connell, 1987). Growth rate was obtained using Annual (Ct) = Af − Ai, in which Ct is the total growth; Ai = Initial area; Af = Final area (Af = area at T5 and Ai = area at T0). Bimonthly growth between times was defined as Cx = Ax − A_(x−1)_, in which x = time and C = growth. Increase in percentage coverage was calculated as %C = (Ct × 100) / Ai. Increase in the number of polyps was also verified. Total increase in the number of polyps was estimated using (I_t_) = P_f_ − P_i_, in which P_f_ = number of polyps at T5; P_i_ = number of polyps at T0. Bimonthly increase in polyps between periods was calculated using (I_x_) = P_x_ − P_(x-1)_. A total of 1,440 measurements and 720 polyp counts were taken during the study.

#### Fecundity

Three colonies of each species were collected monthly from November 2015 to October 2016 (*N* = 36) at depths of 3 to 6 m to verify the periods of greatest reproductive activity of the species. Colonies were fixed in 10% formaldehyde for later polyp dissection. Two central polyps from each colony for each species (*N* = 6, per month) were chosen and separated for dissection. The colonies were decalcified in 10% formic acid solution and 5% formalin solution. The largest diameter of the oral disc and the distance between the oral disc and the base of the polyp were measured with a caliper to calculate the polyp area and correlate it with the number of oocytes per polyp area (cm²), *i.e*., fecundity (Hall & Hughes, 1996). Polyp area was obtained by calculating the area of the ellipse, in which: S = smallest radius × largest radius × 3.14. The polyps were observed under a stereo microscope to visualize the oocytes. Fecundity rate was monitored during the same period of growth rate sampling. Colonies of three species were collected by visual differentiation mainly based on the size, distance and type of growth of the polyps, as well as the color of the tissue and tentacles, following Bastos et al. (2022). Authorization to collect biological material in Arraial do Cabo was granted by ICMBIO (SISBIO no. 51.094).

#### Settlement and recruitment

Ten 20 by 20 cm quadrat at least 40 cm apart on vertical rocks were scraped to observe larvae settlement from November 2015 to October 2016. The scraped areas were chosen at random in a site where sun coral occurs, including coral patches or aggregations of the genus, and tag identified for monitoring. Every 15 days, we dove to observe the occurrence and count of newly settled larvae. After counting, these areas were scraped with a steel brush and sponge to remove individuals and any other organisms found there. The substrates were scraped to avoid competition with other biofouling and the observation of new larvae settled during this period. Twenty-four dives were carried out over the course of a year to acquire data and collect information on planulation periods.

After this period of observation, the same areas were maintained without intervention and photographed after two years to estimate the percentage cover of organisms and to identify species or groups within the quadrat. These field photos were analyzed using the image analysis program Coral Point Count with Excel extensions 4.1 software (CPCe 4.1) (Kohler & Gill, 2006). To estimate the percentage cover and species composition of the fouling community in quadrats, a grid with 92 evenly distributed points was superimposed on the image to count the organisms and the percentage cover of benthic organisms. Percentages of sediment and empty space were also recorded.

#### Temperature

Water temperature was recorded every 1 h by two sensors, with an autonomy of 2 to 3 months, at an average depth of 3 m-Data logger DS 1921 L-F5, iButton, Maxim Inc. (Thermochron^®^ iButtons^®^; Columbia, MD, USA) throughout all the years sampled.

### Statistical analysis

Descriptive statistics were applied and the data were first analyzed for normality using the Shapiro-Wilk test. Subsequently, all the data were analyzed by repeated measures analysis of variance (ANOVA) in the R language and environment for statistical computing in R Studio (RStudio Team, 2015). We performed a pairwise comparison to examine significant sources of variation with Tukey’s multiple comparisons of means with 95% confidence level. Correlation between biological and environmental factors was investigated by Pearson’s correlation. Thermal amplitude was calculated based on the average monthly maximum and minimum temperatures.

## Results

### Growth

#### Colony area

Coverage area of the colonies in relation to time presented highly significant differences between T3 (18.63 ± 9.60 cm²) and T0 (15.83 ± 9.31cm²; ANOVA F = 5.267; *p* = 0.0225). The differences were even greater for T4 and T5 (19.55 ± 9.47 cm²; ANOVA F = 9.372; *p* = 0.002; 20.07 ± 9.171cm²; ANOVA F = 16.382; *p* = 7.0 × 10^−5^, respectively) compared to T0. Notably, the high variance of the means is due to the sampling of different-sized colonies. For individual colonies, these significant differences in size increase over time are maintained (Table 1).

**Table 1:** Average coverage area of three morphotypes of *Tubastraea*. The average coverage area of three morphotypes of *Tubastraea* over a year (Nov/2015 to Oct/2016). T0 = November; T1 = January; T2 = April; T3 = June; T4 = August and T5 = October.

Species	Colony area/cm² (mean + sd)					
	T0	T1	T2	T3	T4	T5
*Ta*	16.56 ± 9.30	17.10 ± 9.44	18.12 ± 9.62	19.24 ± 9.40	19.93 ± 9.36	20.91 ± 9.02
*Tc*	15.99 ± 10.34	16.77 ± 10.47	17.76 ± 10.68	18.77 ± 10.69	19.75 ± 10.62	20.83 ± 10.26
*Ts*	15.24 ± 8.07	14.84 ± 7.88	16.71 ± 8.13	17.89 ± 8.78	18.94 ± 8.53	20.24 ± 8.33
Total	15.93 ± 9.22	16.24 ± 9.30	17.53 ± 9.47	18.63 ± 9.59	19.55 ± 9.47	20.07 ± 9.17

**Figure 2:**
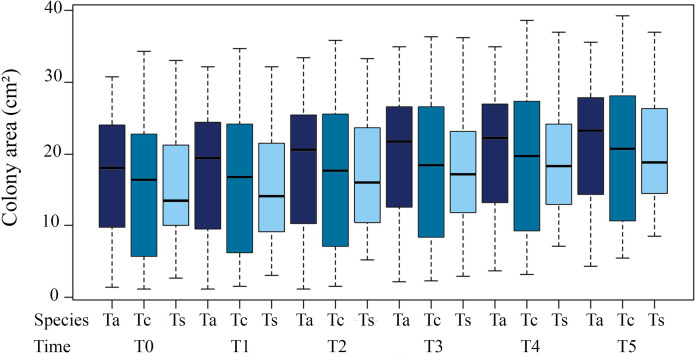
Average coverage area (cm²) of three morphotypes of *Tubastraea* colonies from November 2015 to October 2016. The vertical columns represent the average colony areas of each species over time. Tc, *T. coccinea*; Ta, *T. aurea*; Ts, *Tubastraea* sp.

*Tubastraea* sp. showed the greatest difference in coverage area from the first to the final sampling period (T1 to T5), with the greatest increase in the final coverage area (Fig. 2).

**Figure 3:**
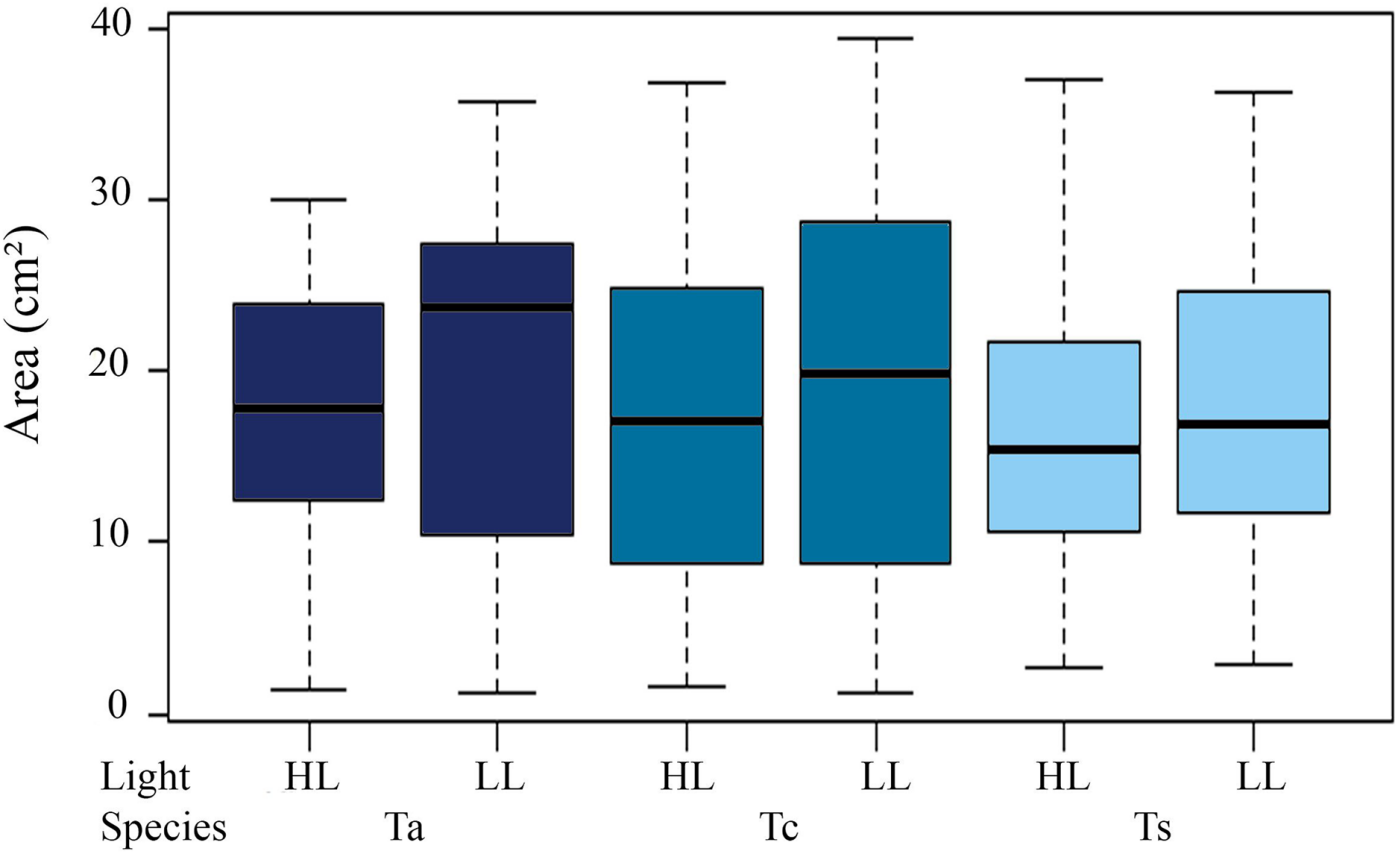
Total annual growth in cm^2^ of all measured colonies of each morphotype between different light levels (LL and HL). Ta, *T. aurea*; Tc, *T. coccinea*; Ts, *Tubastraea* sp.

Mean initial size of the three *Tubastraea* in light environments were higher in LL than HL. The LL values (mean ± SD) were 17.9 ± 5.11 cm² (*T. aurea*), 16.66 ± 7.36 cm² (*T. coccinea*), and 14.38 ± 6.33 cm² (*Tubastraea* sp.) and HL were 15.1 ± 4.49 cm², 15.31 ± 7.71 cm² and 16.1 ± 7.54 cm² for *T. aurea*, *T. coccinea*, and *Tubastraea* sp, respectively. No significant differences were found between initial and final colony area measurements (*p* > 0.05). We observed significant differences for mean values of *T. aurea* comparing LL × HL light levels (ANOVA F = 5.034; *p* = 0.025) and between species: *T. coccinea* × *Tubastraea* sp. (ANOVA F = 8.490, *p* = 0.0039) and *Tubastraea* sp. × *T. aurea* (ANOVA F = 0.301; *p* = 0.039) for LL samples (Fig. 3).

#### Growth rate

**Figure 4:**
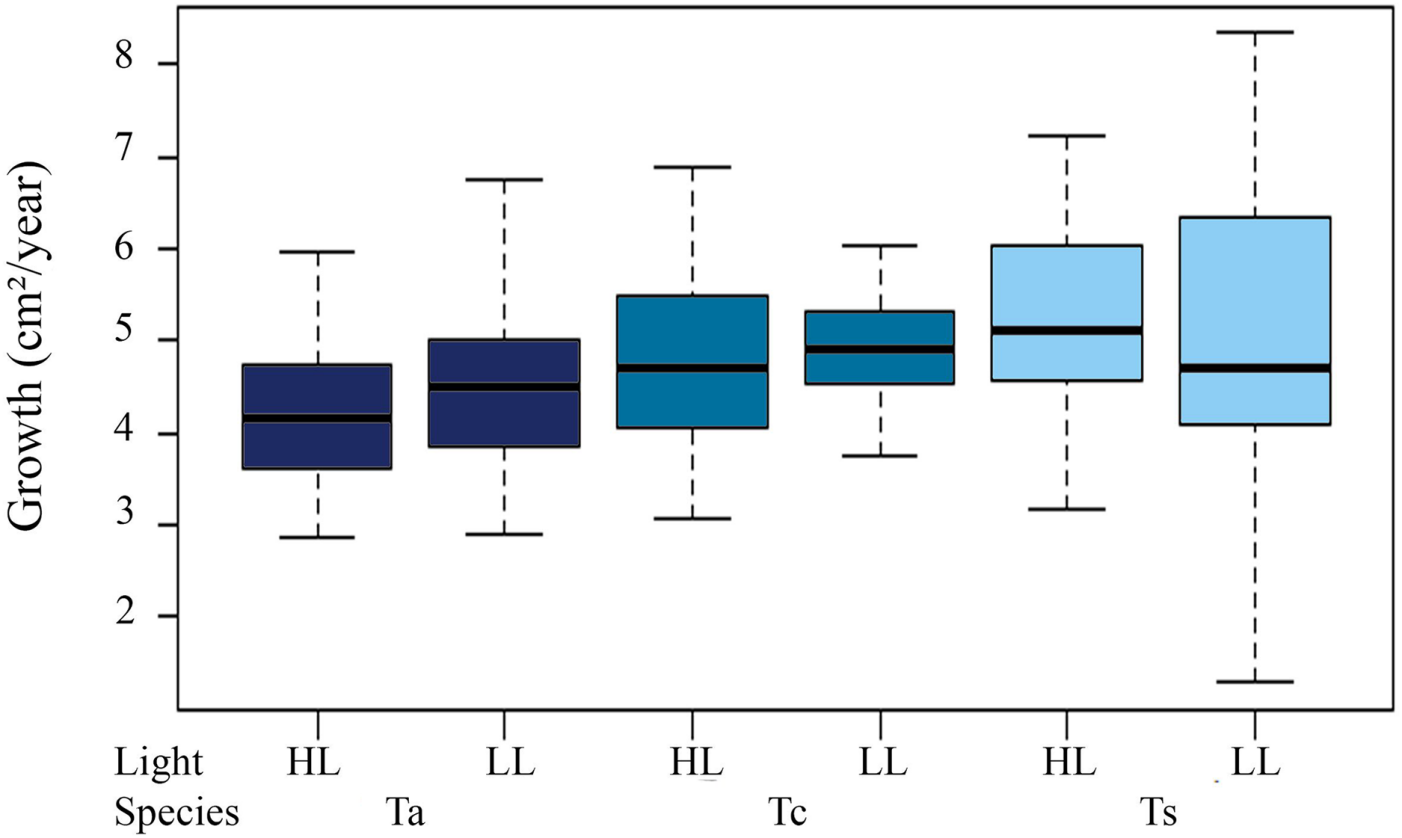
Growth area in two different light levels by morphotypes. Annual growth area in two different light levels by morphotypes under low light irradiance regime (LL) and high light (HL). Ta, *T. aurea*; Tc, *T. coccinea*; Ts, *Tubastraea* sp.

Annual total growth of all the species was equivalent to a coverage area of 567.74 cm² (Table 2). The greatest growth over time was observed between T1 and T2, *i.e*., C2 = 155.06 cm², representing a total increase in size of 15.36% for this period, showing a highly significant difference from the initial growth (C1 = 49.24 cm², ANOVA F = 20.238, *p* = 1.06 × 10^−5^). Among species, the highest Ct was observed for *Tubastraea* sp., with an average growth of 5 cm² per colony per year. *Tubastraea coccinea* showed an average growth per colony of 4.84 cm^2^/year, while *T. aurea* grew 4.35 cm^2^/year, presenting the lowest growth compared to the other species. Nonetheless, *Tubastraea* sp. showed the greatest overall increase in area occurring in T2, responsible for the greatest total increase in cover in all periods. The average growth rate of *T. aurea* was 4.46 cm^2^/year in LL and 4.24 cm^2^/year in HL. For *T. coccinea*, the growth rate was 4.92 cm^2^/year in LL and 4.77 cm²/year in HL. For *Tubastraea* sp., the averages were 4.83 cm^2^/year and 5.17 cm^2^/year in LL and HL, respectively (Fig. 4).

**Table 2:** Growth area in cm² along the times.

Time	Growth area (cm²)					
	*T. aurea*	*T. coccinea*	*Tubastraea* sp.	All species	LL	HL
C1	22.00	31.23	−15.99	37.25	17.69	19.55
C2	40.67	39.71	74.67	155.06	83.82	71.24
C3	44.83	40.01	47.16	132.01	53.14	78.86
C4	27.57	39.67	42.16	109.41	68.15	41.25
C5	39.02	43.15	51.82	134.00	61.26	72.73
Ct	174.09	193.77	199.82	567.74	284.07	283.67

**Note:**

Growth area in cm² along the times, in each morphotypes of Tubastraea during a period of one year, in the different light levels and the total in each of them. The “C” times are equivalent to a period of approximately 2 and a half months.

#### Polyps

Total increase in polyps (It) in one year was 253, 392, and 230 polyps for *T. aurea, T. coccinea* and *Tubastraea* sp., respectively. Significant differences in total increase occurred only between *T. aurea* and *T. coccinea* (ANOVA F = 7.820, *p* = 0.0065) (Table 3). We observed a strong contribution from *T. coccinea* in all intervals except I2, with the greatest difference occurring in *Tubastraea* sp. compared to the other species.

**Table 3:** Polyp increment between times for each three *Tubastraea* morphotypes.

Species	Number of polyp increment (un)				
	I_1_	I_2_	I_3_	I_4_	I_5_
*Ta*	41	61	26	57	68
*Tc*	86	35	67	91	113
*Ts*	20	66	51	21	72
Total	147	162	144	169	253

**Note:**

Polyp increment between times for each of three *Tubastraea* morphotypes and the total between the times, where intervals (I) represent the difference in the number of polyps between time T and time T-1.

Comparing the light levels (LL × HL) in total average increase (polyps/year per colony), we found no significant difference within each species (*p* > 0.05), except for *T. coccinea* (*p* = 0.04). Comparing the species between light levels in the sampling times, only *T. coccinea* × *Tubastraea* sp. showed a significant difference for LL (ANOVA F = 5.317, *p* = 0.026). Both species had a large increase in the number of polyps by extra-tentacle budding through the coenosarc.

In each time period, we observed significant differences only at T1 between *T. coccinea* × *Tubastraea* sp. (ANOVA F = 8.111, *p* = 0.0056) (Table 4). Within each species group, all *Tubastraea* species showed differences between sampling times. For *T. aurea* and *Tubastraea* sp. the differences occurred at initial measures until T3, T4 and T5. (Table 5).

**Table 4:** Polyp number of *Tubastraea*.

Specie	Polyps number (mean + sd)					
	T0	T1	T2	T3	T4	T5
*Ta*	9.21 ± 5.60	10.20 ± 5.48	11.70 ± 5.57	12.10 ± 5.56	13.50 ± 5.82	15.22 ± 5.50
*Tc*	10.27 ± 4.75	11.70 ± 5.37	12.45 ± 5.46	14.07 ± 5.70	16.31 ± 4.38	18.37 ± 5.31
*Ts*	8.60 ± 4.73	8.52 ± 3.81	10.17 ± 3.75	11.45 ± 4.13	11.87 ± 4.23	13.67 ± 4.11
Total	9.20 ± 5.08	10.20 ± 5.09	10.85 ± 5.00	12.01 ± 5.15	12.63 ± 4.85	14.20 ± 5.18

**Note:**

The average number of polyps and standard deviation of each of the three species of *Tubastraea* over a 1-year period.

**Table 5:** ANOVA results of *Tubastraea* population dynamics. F statistics and significance levels from repeated measures factorial ANOVA on parameters of population dynamics of three *Tubastraea* morphotypes along a year. Time and species presented interaction within in all evaluated parameters and specie also showed a slight interaction with time or light regime. Significance codes: *p* < 0.1, *p < 0.05, **p < 0.01, ***p < 0.001.

ANOVA effects	*Growth*	*Polyp*
Time	0.00797**	9.07 × 10^−15^***
Species	2 × 10^−15^***	0.00476**
Light regime	2 × 10^−15^***	0.08761
Time × Specie	0.94550	1.4 × 10^−7^***
Time × Light	0.89000	0.5817
Specie × Light	0.00488**	0.62242
Time × Sp × Light	0.99300	0.36201

### Fecundity

**Figure 5:**
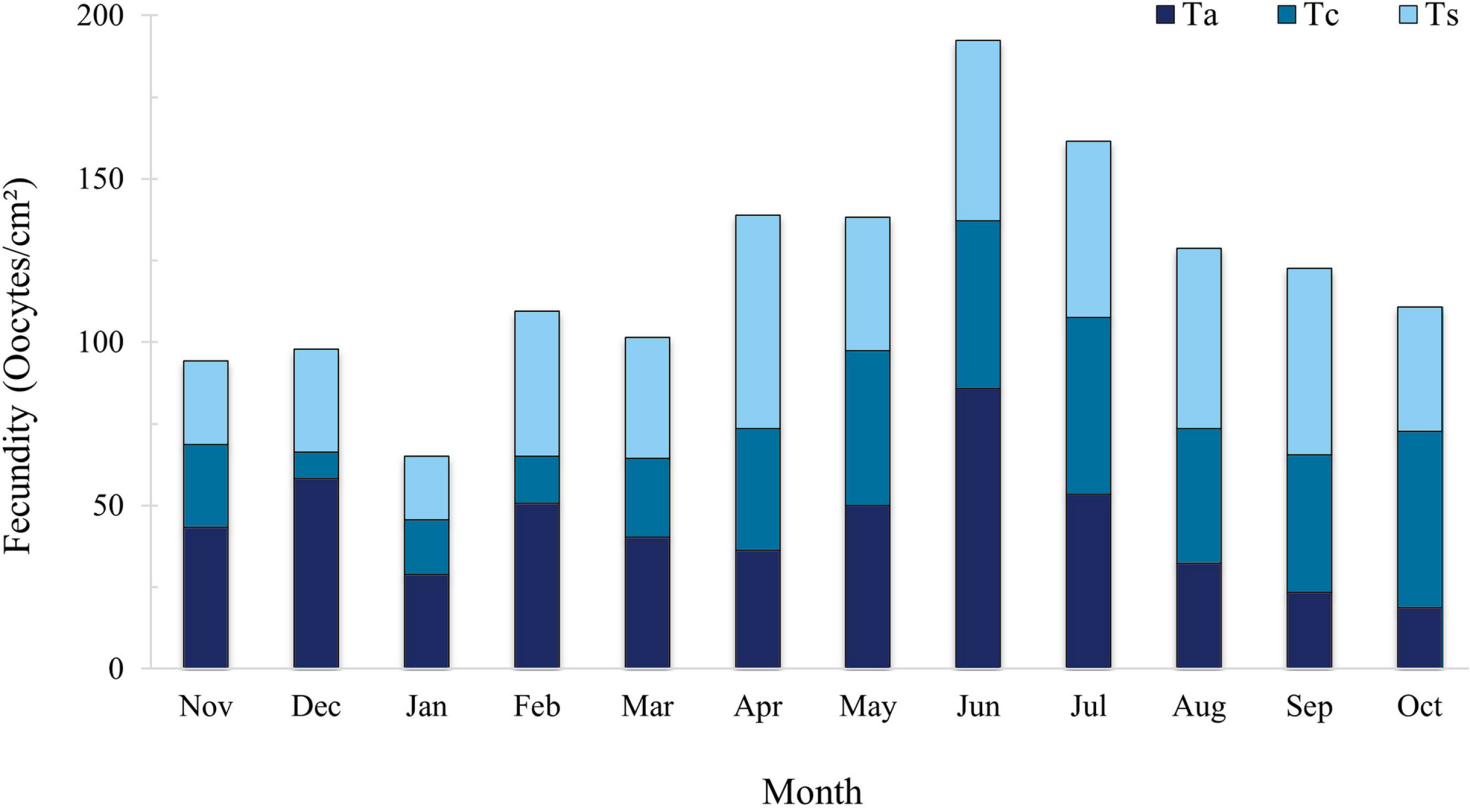
Fecundity rate (oocytes/mm^2^) of three *Tubastraea* morphotypes sampled between November 2015 and October 2016. The highest total fecundity rates occurred in June and July, and the species with the highest rates for Ta and Ts, with the month of June being Ta. Ta, *T. aurea*; Tc, *T. coccinea*; Ts, *Tubastraea* sp. Each vertical bar represents the three morphotypes’ total fecundity rate (oocytes/cm²) over time. The lines show fecundity data for each morphotype separately.

The total mean for the entire sampling period was 40.58 ± 3.22 oocyte/cm². *Tubastraea aurea* and *Tubastraea* sp. showed the highest mean fecundity rates, with 43.50 ± 5.23 and 43.60 ± 4.04 oocytes/cm², respectively, without significant differences between them. *Tubastraea coccinea* had the lowest mean rate among the species, 34.64 ± 4.72 oocytes/cm².The highest total fecundity rates, adding up the three species observed, occurred in November and December, with 94.24 and 97.88 oocytes/cm²; the lowest fecundity rates occurred in June and July, with means of 64.11 ± 4.46 and 53.80 ± 0.08 oocytes/cm² (Fig. 5). It was impossible to differentiate the few embryos and larvae that appeared in the samples, so we only considered those oocytes that were clearly identifiable and classifiable according to their developmental stage.

### Settlement and recruitment

**Figure 6:**
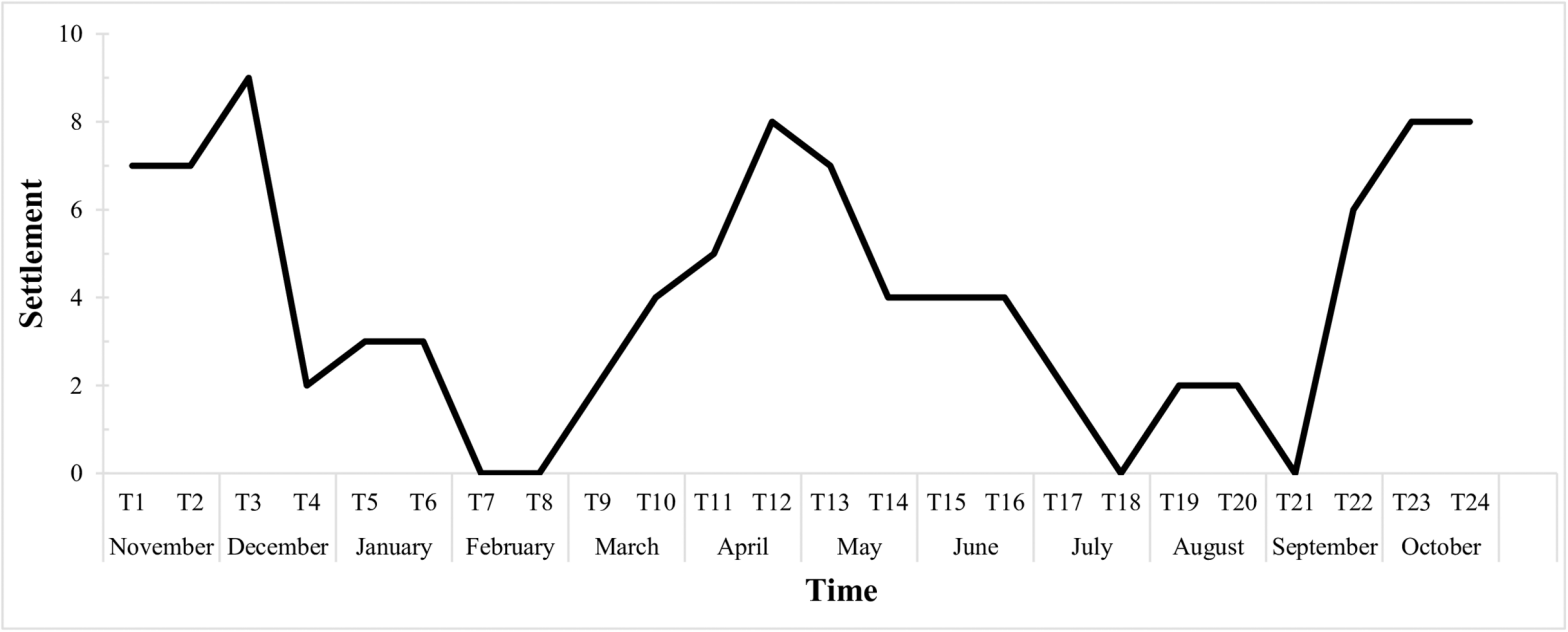
Biweekly average settlement rate of *Tubastraea* spp. larvae from Nov 2015 to Oct 2016 in 10 scraped areas on the rocky shore. The line represents the settlement rate over the sample year. Each time is 15 days long.

The graph below (Fig. 6) shows the fortnightly settlement values, where T1 represents the first sampling in November 2015. The mean fortnightly settlement rate was 4.04 ± 2.88 larvae/quadrat, with peak values in November and December (T2 and T3), April (T11 and T12) and October (T23 and T24). The minimum rates occurred in February (T7 and T8), with no settlement, then in July (T17 and T18), and then from late August to early September (T20 and T21).

**Figure 7:**
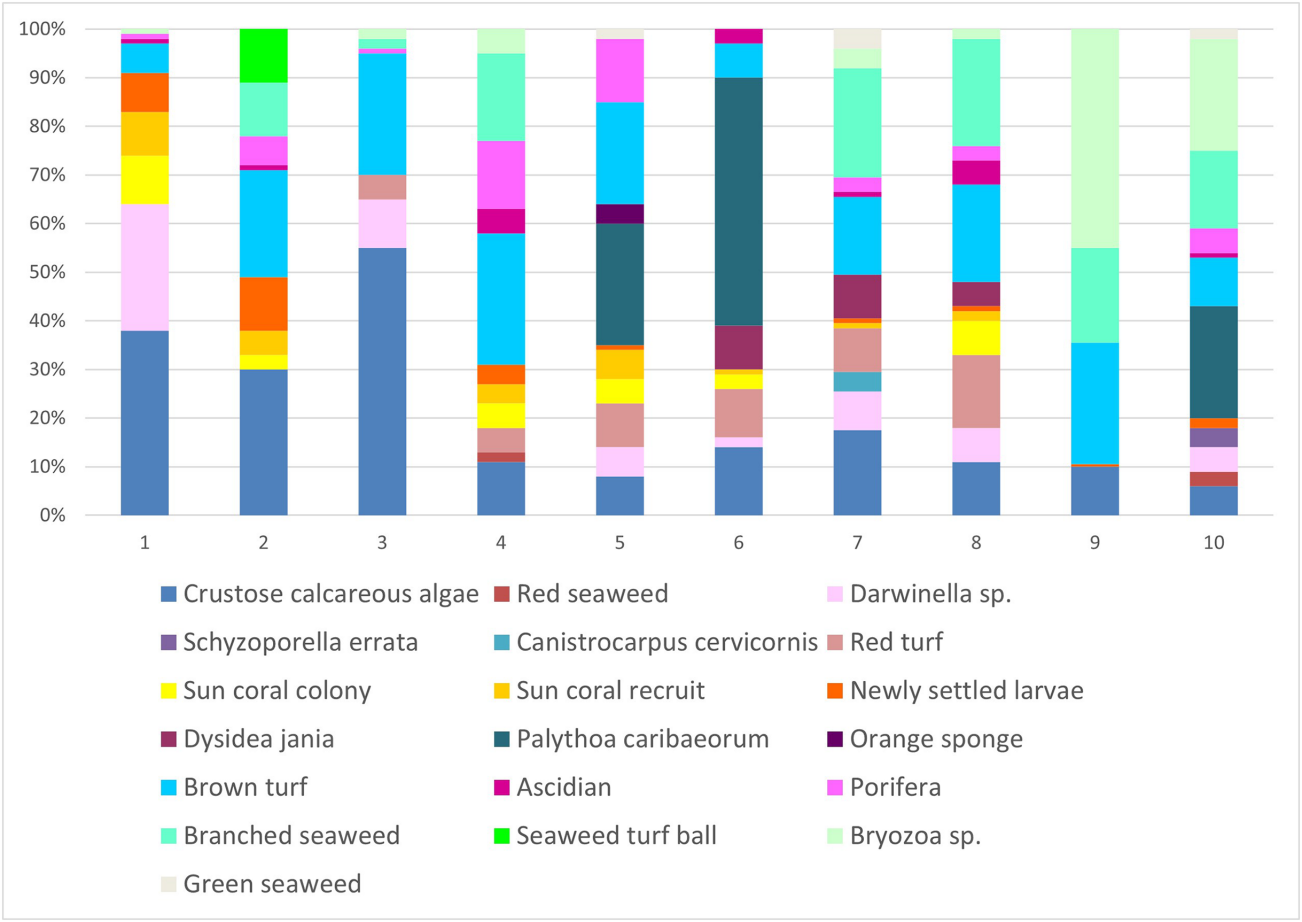
Percentage of coverage of organisms found in the scraped areas numbered from 1 to 10 in the place of occurrence of sun coral. Not all individuals were identified, so they were given names according to their shape or color. Each of the colors represents each of the 19 groupings of organisms identified during collection. The x-axis represents the proportion (%) of each sampled group.

In scraped areas on rocky shores, 2 years later, the newly-fixed larvae of *Tubastraea* corals covered a 2.85% area, whereas recruits and colonies accounted for 2.8 and 3.3% of the coverage area, respectively. Fixed larvae, recruits and sun coral colonies accounted for 8.9% of the total coverage area sampled. Encrusting calcareous algae, brown turf algae, branched algae, *Palythoa caribaeorum*, *Bryozoa* sp., *Darwinella* sp. and red peat were the organisms that occupied the most space in all quadrats. Calcareous algae occurred in 20% of the areas studied. (Fig. 7). Nine of 10 scraped quadrats contained newly settled larvae and/or recruits and/or sun coral colonies. Together, all the sponges seen at the intersection points amounted to 13.8%, algae covered 37.1%, bryozoans 8.6%, cnidaria (*Palythoa caribaeorum*) 9.9% and ascidians 1.7%.

### Temperature

The temperature values sampled over the course of a year were analyzed. A graph with all the data recorded by the iButton temperature sensors and their daily moving average can be found in the Supplementary section (Fig. SA). Annual average temperature was 21.84 ± 1.73 °C (mean ± SD), with maximum temperature of 26.25 °C and minimum of 14.5 °C. March and April, 2016 had the highest temperatures and January, 2016, the lowest. Temperature between seasons was similar, averaging 22.06 °C in spring, 22.17 °C in summer, 23.13 °C in fall and 22.17 °C in winter.

We consider that values below 20 °C refer to the influence of upwelling waters-from May to September 2016 no upwelling event was detected, *i.e*., in months after the end of summer and early spring, precisely the months when the phenomenon occurs in the region, thus demonstrating an atypical year.

By analyzing these data, we observe some seasonal patterns in the temperature variation (Fig. SB). A broader plot of data demonstrates a wide and differentiated temperature rate (Δ°C) for the months of late spring to late summer. Meanwhile, the data for the fall and winter months have much less variability than the months influenced by upwelling.

Based on the previous results, we plotted the data to visualize only the data on thermal amplitude for the same sampling period (Fig. SB). We observed a pattern of temperature variation throughout the year, with high variations during the summer, reaching 10 °C in March.

### Growth *versus* fecundity and thermal amplitude

**Figure 8:**
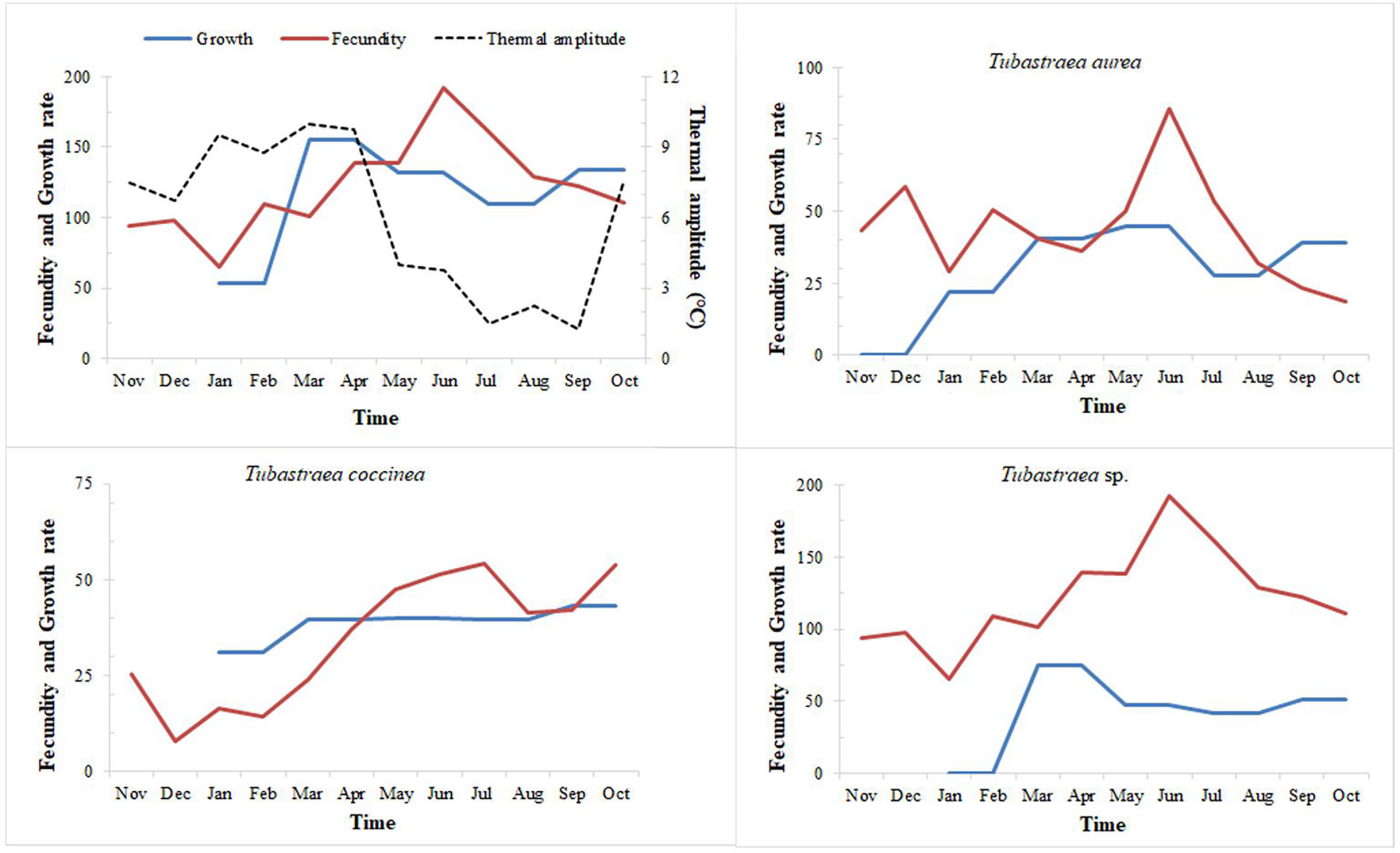
Fecundity and growth rates of three morphotypes of *Tubastraea*. Fecundity and growth rates of *Tubastraea* genus in Arraial do Cabo and each of the three morphotypes of *Tubastraea* over the period of 1 year. The right axis represents the thermal amplitude over the same period (dashed line). In periods of more constant temperature, growth and fecundity rates show a clear pattern. Fecundity in oocyte/cm^2^ and growth rate in cm^2^.

When we relate the temperature data to growth rates (*i.e*., growth in area), we can see that the greatest overall growth occurred from January to March, a period which also showed the greatest variation in temperature over the year sampled (Fig. 8). During this period, we also observe the greatest increase in area for *Tubastraea* sp. The greatest increases at different light levels were also observed in the same period, for both LL (January–March) and HL, especially for *Tubastraea* sp.

When relating growth with fecundity rates throughout the year, we note a pattern of behavior: when fecundity rates are highest, growth decreases, increasing again when the number of oocytes per area begins to decrease. When we have the lowest fecundity rates in the summer months, we see an abrupt increase in growth from February onwards (see Fig. 8). Even so, by plotting thermal amplitude lines, we observe that high temperature variation does not favor the fecundity of *Tubastraea* colonies, whereas lower temperatures favor growth. However, temperatures below 16 °C may be acting as a barrier to growth (see graph with maximum and minimum temperatures, Fig. SB).

The more constant temperatures seem to favor higher fecundity rates or the high temperatures that precede months of high fecundity may be acting as a trigger or as a catalyst for the next fecundity period. Our correlation tests (Pearson’s r) shows a weak inverse correlation between fecundity and thermal amplitude (r = −0.232). We found a similar correlation between growth and thermal amplitude (r = −0.158).

## Discussion

Arraial do Cabo’s sun corals demonstrated favorable growth at lower and higher average temperatures, which is considered an interspecific variation. *Tubastraea* sp. species reached the highest growth rate and total growth from January to March, the period with the greatest temperature variation. January and February also had the lowest averages of the year, 20.8 °C and 20.9 °C, respectively, and in March, the average increased to 22.2 °C. The contribution of *Tubastraea* sp. was especially relevant in that season. On the other hand, *Tubastraea coccinea* colonies grew more in lower thermal amplitude, when temperatures varied from 20 °C to 23 °C (August and September), manifesting a possible reproductive behavior with distinct strategies between both species. The increase in water temperature in April and May, reaching almost 26 °C, boosted the growth of *Tubastraea aurea*, with temperature averages higher than the annual average of 21.8 °C. Considering that an annual temperature cycle is repeated over the years, the supply of nutrients in periods of upwelling related to the higher densities of zooplankton in Arraial do Cabo (Valentin, 1984a) contribute to the growth area of *Tubastraea* species. This corresponds to our finding that there was greater growth in upwelling months than in other months.

The study by Mizrahi (2008) found a positive relationship between increasing temperature and growth in *T. coccinea*, in which the coral increased by 4.59 cm^2^ year^−1^ in a location at 21.6 °C, while at 20.8 °C growth was 1.14 cm^2^ year^−1^. All species showed greater growth at temperatures above 21 °C, emphasizing *T. coccinea* and *T. aurea* under those conditions, corroborating our results about *T. aurea*. Some studies have shown a correlation between a certain degree of increase in temperatures and coral cover rates. According to Lough & Barnes (2000), a 1 °C increase in the sea surface increment the annual rate of calcification and the average annual extent of massive corals of the genus *Porites*. Once again, the average growth of *Tubastraea* is much higher than other scleractinian corals, such as the massive coral of the genus *Siderastrea*, with a linear growth rate of 2.5 mm per year, according to Lins de Barros & Pires (2006), while *Tubastraea* reached 4.73 cm^2^/year in this study. Vermeij (2005) considered *Tubastraea* to have accelerated growth, describing a growth of 3.02 cm^2^ year^−1^ in Curaçao. Low temperatures also affect the growth of sun corals (Saá et al., 2019), when *T. coccinea* colonies showed no activity or growth at temperatures below or equal to 16 °C and died in these colder conditions after the sixth day of the experiment. Thus, temperature affects the physiology of individuals in several ways and can harm their competitive interactions as well as their ability to exploit resources (Kordas, Harley & O’Connor, 2011; Reuman, Holt & Yvon-Duroche, 2014). Higher temperatures benefit calcification; however, very high temperatures, such as 28–31 °C, demonstrate the dissolution of calcium carbonate, according to the authors.

*Tubastraea* corals preferably inhabit shaded areas, as they do not present symbiosis with photosynthetic algae for energetic supply. Moreover, despite being more abundant in vertical, inclined, and negative areas, colonies in positive areas are observed nowadays. As such, these colonies, which are exploring new niches, were considered to compare the life history of these organisms with those living in shaded areas. Mizrahi (2008) reported a higher growth rate of *T. coccinea* in sites with higher luminosity and temperatures as well lower growth in places with low light and temperatures. In our study, we found no significant differences in growth at different light levels; however, *T. coccinea* and *T. aurea* grew more in areas with less light, showing that they are more adapted to shaded areas than lit areas. In fact, the light regime was a relevant factor in growth of *T. aurea*. We observed the opposite for *Tubastraea* sp. colonies in areas with a greater incidence of direct light, which grew more compared to those in shadier areas, being more common in illuminated areas. Moreover, Mizrahi (2008) showed that temperature was the main factor responsible for reducing the colonization process of this species in Arraial do Cabo. Batista et al. (2017) showed that lower seawater temperatures, due to upwelling phenomena, probably control the rapid expansion of *T. coccinea* in Arraial do Cabo Bay.

The increase in polyps followed the growth pattern. More polyps appeared in the low light regime for *T. aurea* and *T. coccinea*, while for *Tubastraea* sp., more polyps sprouted during the high light regime. *Tubastraea coccinea* had the highest number of polyps growing in 1 year, averaging 8.1 polyps/year. Morphological studies demonstrated that *T. coccinea* has more polyps per colony area than *T. aurea* and *Tubastraea* sp. According to Bastos et al. (2022), *T. coccinea* presents polyps with smaller calyx diameters than the others, and its skeleton is not as robust as that of *T. aurea*. Morphology may explain this result because the type of dendroid growth of *Tubastraea* sp. showed a lower increase in polyps. Additionally, corals can reproduce asexually and by budding to expand the colony. This is a common form of colony enlargement among sun corals, in which new polyps grow from an old polyp or from the oral disc of a parental polyp. Fragmentation can also occur by forming two new colonies (Campbell, 1983), but this has not been observed in the field.

Regarding fecundity, we suggest that *Tubastraea* corals may release larvae constantly. However, two periods of peak reproduction are considered spawning periods: one in spring from October to December, and another in autumn from April to June, based on settlement and fecundity findings. Similar to our outcomes, De Paula, Pires & Creed (2014) recorded greater reproductive effort; from September to December and another period from February to May and Crivellaro et al. (2020) in September and early spring. The months with the greatest settlement were almost 1 °C warmer than those with little or no larval settlement. In autumn, high fecundity and settlement rates were recorded, and the average temperature was the highest among the seasons, at 23.13 °C. Fecundity rates remained high in winter, with an average temperature of 22.17 °C. We can suggest an affinity between higher water temperature averages and greater reproductive activity of *Tubastraea* corals in Arraial do Cabo. This suggests low temperatures as a limiting factor in the population dynamics of *Tubastraea*.

The invasive corals produce gametes and larvae continuously due to the occurrence of larvae in at least one of the samples every month of the year. Reproductive peaks related to fecundity occurred from April to July, and those related to larval recruitment occurred from April to June and October to December. A directly proportional pattern of settlement rate and thermal amplitude was observed from April onwards. However, in the previous period, we observed minimum settlement rates from January to March, which also corresponds to lower temperatures. In the recent study by Mizrahi et al. (2023), the authors described a significant decrease in the release of sun coral larvae at temperatures below 24.5 °C and an increased release at higher temperatures (≥24.5, <27.2 °C) and greater water turbidity. During summer, we recorded low fecundity and little or no settlement; however, we observed greater growth.

Data on fecundity, growth rates, and tissue energy content allow for estimate the relative caloric investment in the colony’s growth and reproduction processes by planulation (Richmond, 1987). Richmond studied the scleractinian coral *Pocillopora damicornis* in the eastern Pacific (Panama). The coral did not release larvae for two years, and during this period, it showed a higher linear growth rate of 3.6 to 6 cm per year. Colonies of this coral in two distinct areas allocated similar amounts of the colony’s caloric content to biomass production; however, in one region, most of this energy was represented by planulation, and another was allocated to colony growth and subsequent fragmentation. The fecundity rate in Arraial do Cabo was much higher than in Ilha Grande Bay, Rio de Janeiro, being around three to four times higher than that of *T. coccinea* (10 oocytes/cm²) and an even greater difference in relation to *T. tagusensis* (2.68 oocytes/cm²) (De Paula, 2007). From March to May, there was an increase in the average water temperature, with April reaching the highest average of the year, 24.13 °C, which was related to peaks in larval settlement in the field. The scleractinian coral larval settlement has previously been positively related to increasing temperature by Harriott & Fisk (1988), and summer recruitment is related to the spawning of most coral species in the GBR in late spring and early summer. However, Airi et al. (2014) reported a decrease in the reproductive efficiency of endemic zooxanthellate corals with an excessive increase in water temperature of up to 32 °C in the Mediterranean Sea. Fecundity was also related to increased temperature in *T. coccinea* and *Tubastraea* sp. According to Airi et al. (2014), changes in water temperature can alter the physiological function, reproductive production, and demography of marine organisms. Babcock et al. (1986), Oliver et al. (1988), and Harrison & Wallace (1990) suggest that variations in water temperature and photoperiod can regulate the coral reproductive cycle. Different environmental pressures can affect the reproductive strategy of corals in different parts of their geographic distribution (Castro et al., 2006). Thus, we reiterate that low temperatures may be a limiting factor for larvae settlement.

We observed *Tubastraea* larvae, recruits, or small colonies among 19 other identified organisms in the scraped areas. Crusted coralline algae, brown algae, branched algae, *Palythoa caribaeorum*, *Bryozoa* sp., *Darwinella* sp., and red peat were the most common organisms found throughout the quadrat. According to Silva et al. (2022), sun coral larvae demonstrated a preference (63%) for settling in crustose coralline algae on islands off the coast of the city of Rio de Janeiro. The sites where *Tubastraea* spp. were present and more abundant, presented greater diversity, uniformity, and species richness than sites without these exotic corals (Lages et al., 2011). As replacement of species composition is outside the scope of this research, a long-time scale was used to collect biotic data so that variations in species abundance were missed and temporal patterns in substrate occupancy by different dominant species could not be observed. Species richness and diversity in the available sites demonstrate that *Tubastraea* spp. corals co-occur with native organisms and may not negatively affect the development of benthic communities. Its high competitive potential in colonizing new substrates was reflected in the covered area, which was found to be 8.9%. On the other hand, studies in Arraial do Cabo found low abundance and restricted distribution of corals of the genus *Tubastraea* compared to other regions (Batista et al., 2017). In Araújo’s (2023) doctoral dissertation, in the same site of our study, she observed that benthic species richness, diversity, and evenness did not differ between communities invaded and not invaded by sun coral. *Tubastraea coccinea* is increasing its expansion capacity with higher growth rates than those documented approximately 10 years ago in the region (Mizrahi, 2008). As a reproductive strategy of an invasive coral, *Tubastraea coccinea* presents higher annual fecundity estimates compared to the zooxanthellate species described by Harrison & Wallace (1990). The fecundity of *T. coccinea* is comparable to that of other breeding species with notable fecundity, for example, *Porites panamensis* reported by Glynn et al. (1994) in Panama and *Stylophora pistillata* observed by Loya (1976) in the Red Sea, and by Hall & Hughes (1996) on the Great Barrier Reef. Likewise, our study showed high comparative fecundity rates and, therefore, high potential for population increase in the region.

Our study recorded the greatest coral growth ever reported for the region, showing the current adaptation of the studied species are achieving throughout the invasion process. Our findings indicate that these invasive corals are well-established and adapted to the location studied. We can also observe that their reproductive strategies are even more efficient. Furthermore, our results demonstrated an increase in niche, enabling the colonization of positive areas with greater luminosity. Studies of ecological interactions are essential to evaluate the relationship between organisms. Despite this, the lower temperatures in the region affect the reproductive activity of corals, slowing down the process of introduction into the region. On the other hand, the constant changes in the normality of environmental factors, a result of climate change, and the increase in the occurrence of high-water temperatures can facilitate the populational expansion of these invasive corals.

The monitoring of invasive marine corals is crucial for informed decision-making by managers aiming to control and conserve marine biodiversity. Invasive corals can outcompete native species for resources, disrupt ecological balance, and degrade habitats, leading to significant biodiversity loss. By systematically tracking the distribution, population dynamics and impact of these invasive species, managers can develop targeted strategies to mitigate their spread, restore affected ecosystems, and ensure the resilience of marine environments. Additionally, comprehensive data gathered from monitoring programs can inform the development of targeted management plans, support the allocation of resources, and enhance the effectiveness of conservation efforts. Effective monitoring also facilitates early detection and timely response efforts, which are essential for preventing the establishment and proliferation of invasive corals. Ultimately, the integration of monitoring data into management decisions is essential for maintaining the health and resilience of marine ecosystems.

## Supplemental Information

10.7717/peerj.17829/supp-1 Supplemental Information 1 Temperatures recorded hourly over a year at Arraial do Cabo Bay over twelve months between 2015 and 2016. The gray line shows the raw data and the black line represents the average daily temperature (°C).

10.7717/peerj.17829/supp-2 Supplemental Information 2 Annual thermal amplitude (Δ°C) over a year of sampling at Arraial do Cabo Bay, RJ – Brazil. Upper dotted line represents the maximum temperatures while the dotted line below the minimum temperatures. Bold line represents the averages by time.

10.7717/peerj.17829/supp-3 Supplemental Information 3 Growth rate in species under different light intensities in the environment they inhabit.

10.7717/peerj.17829/supp-4 Supplemental Information 4 Fecundity rate in species at two polyps per colony.

10.7717/peerj.17829/supp-5 Supplemental Information 5 Area increase and polyp increment at different follow-up times.

## Additional Information and Declarations

### Field Study Permissions

The following information was supplied relating to field study approvals (*i.e*., approving body and any reference numbers): Field experiments were approved by ICMBIO/IBAMA.

### Data Availability

The following information was supplied regarding data availability: The raw data are available in the Supplemental Files.
