# Supplementary figures and images for: Life history strategy of Tubastraea spp. corals in an upwelling area on the Southwest Atlantic: growth, fecundity, settlement, and recruitment
Source: PeerJ. 2024 Jul 31;12:e17829. doi: 10.7717/peerj.17829 (PMC11297442; doi:10.7717/peerj.17829)

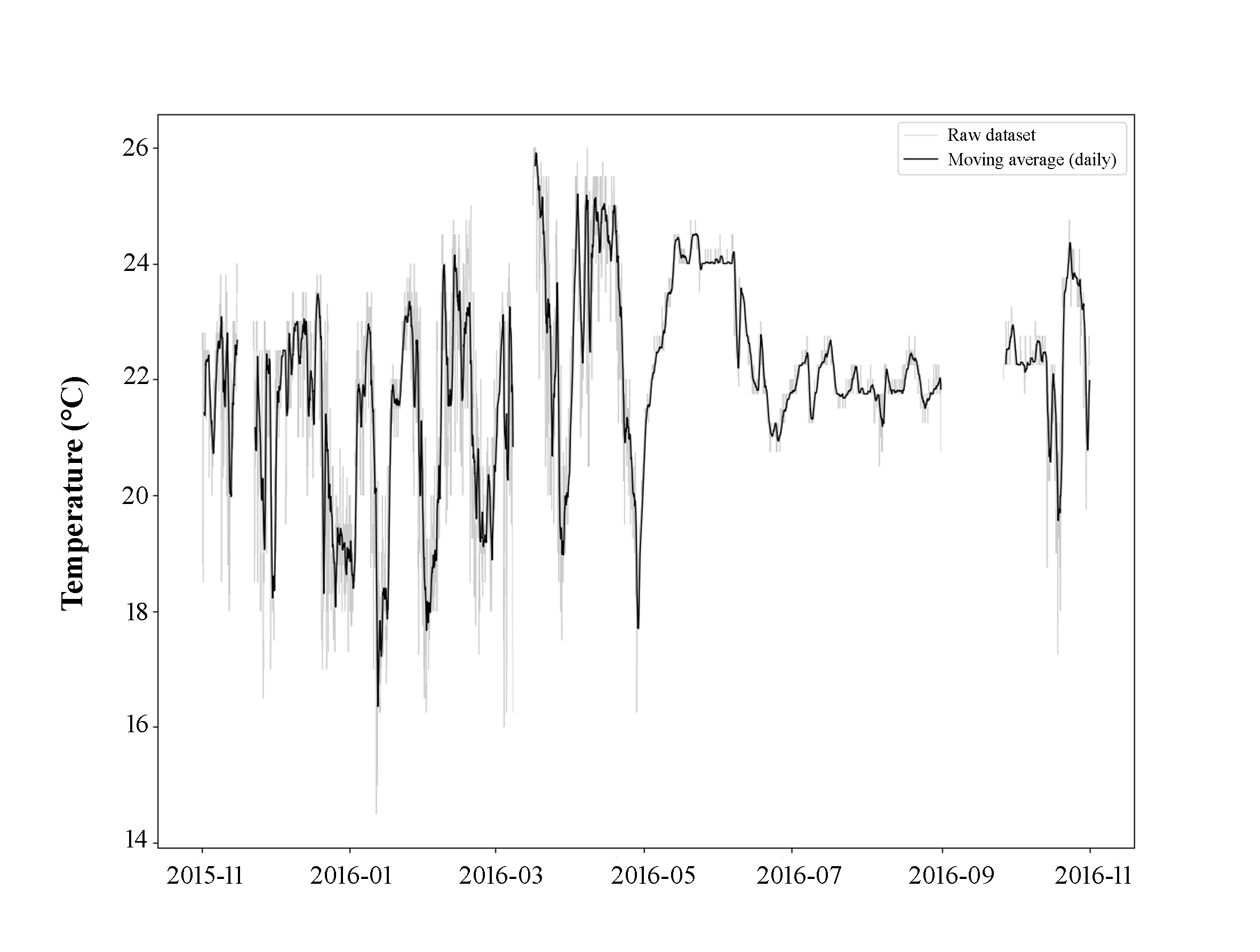

Supplement: Supplemental Information 1 — The gray line shows the raw data and the black line represents the average daily temperature (°C). [file peerj-12-17829-s001.tif]

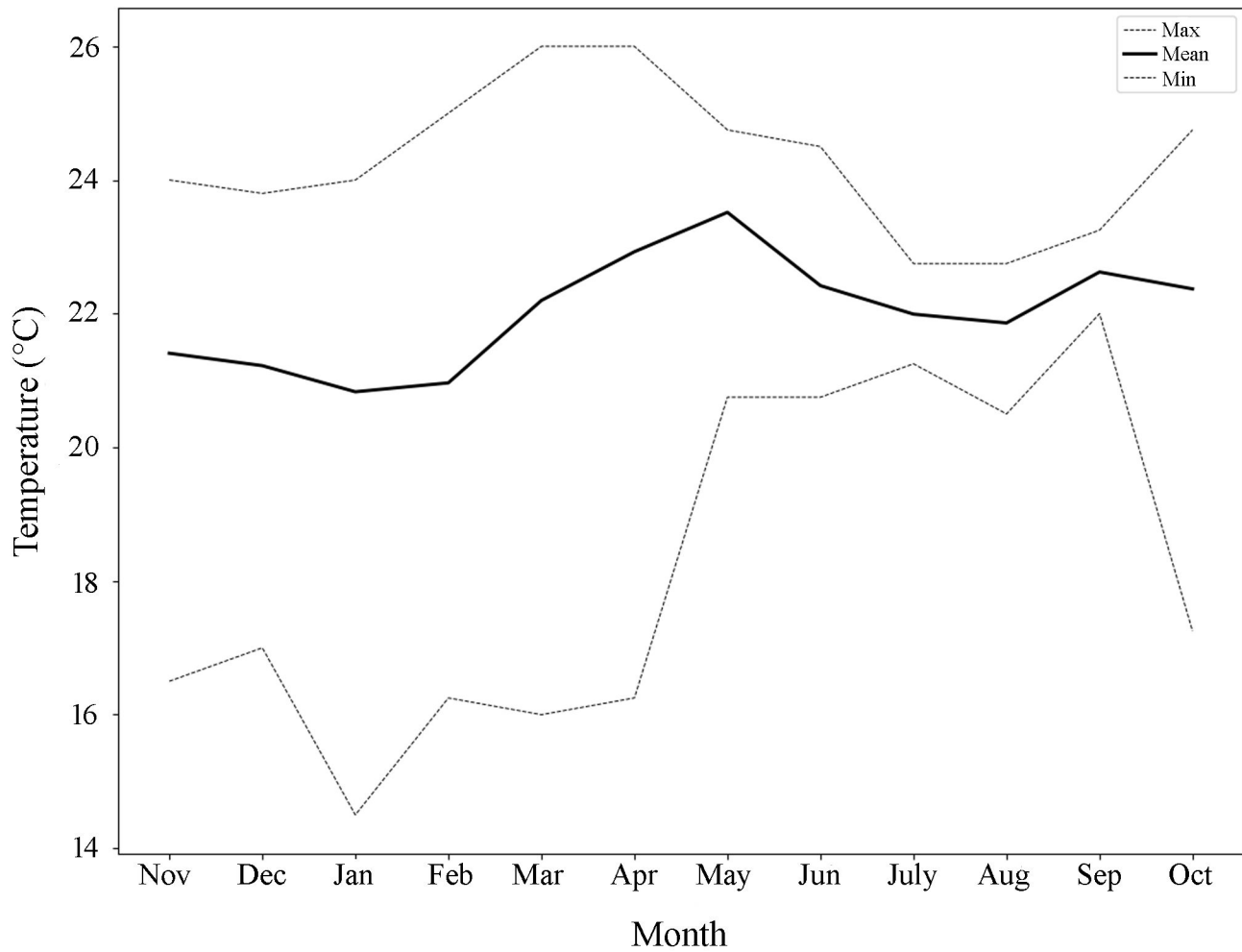

Supplement: Supplemental Information 2 — Upper dotted line represents the maximum temperatures while the dotted line below the minimum temperatures. Bold line represents the averages by time. [file peerj-12-17829-s002.pdf]
